# Haemangioma, an uncommon cause of an extradural or intradural extramedullary mass: case series with radiological pathological correlation

**DOI:** 10.1007/s13244-015-0432-y

**Published:** 2015-09-18

**Authors:** S. H. McEvoy, M. Farrell, F. Brett, S. Looby

**Affiliations:** Department of Neuroradiology, Beaumont Hospital, Dublin, Ireland; Department of Neuropathology, Beaumont Hospital, Dublin, Ireland

**Keywords:** Spine, Haemangioma, Magnetic resonance imaging, Angiography, Pathology

## Abstract

**Abstract:**

Haemangiomas of the vertebrae, usually regarded as having little or no consequence, may display aggressive features, including extension into the extradural space, and cause significant neurological symptoms and signs necessitating treatment. Extraosseous haemangiomas in an extradural or intradural extramedullary location are a rare entity. Here we review our radiologic and pathologic experience of osseous haemangiomas with extradural extension and primary extradural and intradural extramedullary haemangiomas. Magnetic resonance imaging plays a pivotal role in the characterisation of spinal haemangiomas, with typical imaging features including T1 and T2 signal hyperintensity. Atypical and aggressive imaging features are also described. Spinal angiography may be required to differentiate haemangiomas from non-vascular lesions. This is a rare and unusual entity, and should be considered as a differential diagnosis for some extramedullary masses.

***Teaching points*:**

• *Osseous haemangiomas can display aggressive features and cause neurologic symptoms needing treatment.*

• *Haemangioma extension into the extradural space is an imaging feature of aggressiveness.*

• *Extraosseous haemangiomas are a rare but important differential diagnosis for extramedullary masses.*

• *Extraosseous extramedullary haemangiomas most frequently present with progressive myelopathy*.

• *MRI is pivitol in characterising spinal haemangiomas*; *imaging characteristics can vary*.

## Introduction

Haemangiomas are frequently encountered benign vascular tumours in vertebral bodies. In contrast, the clinical and radiologic features of extraosseous haemangiomas are not so well characterised. The nomenclature includes cavernous, capillary, venous or arteriovenous haemangiomas, and is based on the predominant vascular channel abnormality. Additionally, haemangiomas may be classified as high- or low-flow. Magnetic resonance imaging (MRI) plays a pivotal role in determining the characteristics and extent of haemangiomas. Vertebral spinal haemangiomas typically follow a benign course and are regularly regarded as inconsequential, although a small proportion may cause neurological symptoms necessitating active management. This article aims to review haemangiomas of the spine, incorporating both osseous haemangiomas with extradural extension and primary extradural or intradural extramedullary haemangiomas. It correlates radiological features with pathology and discusses current concepts in diagnosis and treatment.

## Methods

**Fig. 1:**
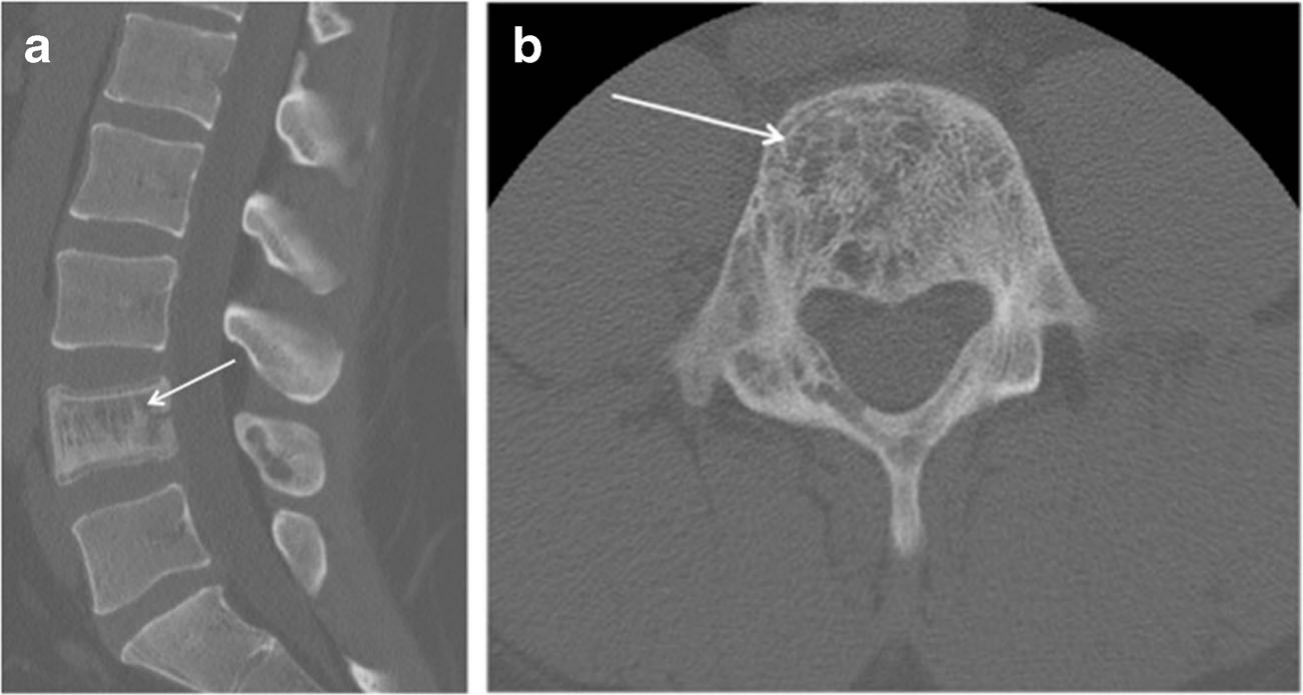
Sagittal CT image (**a**) shows the classical striated appearance of a haemangioma in the L4 vertebral body. Axial CT image (**b**) shows the spiculated, spotted appearance of the lesion in cross-section

**Fig. 2:**
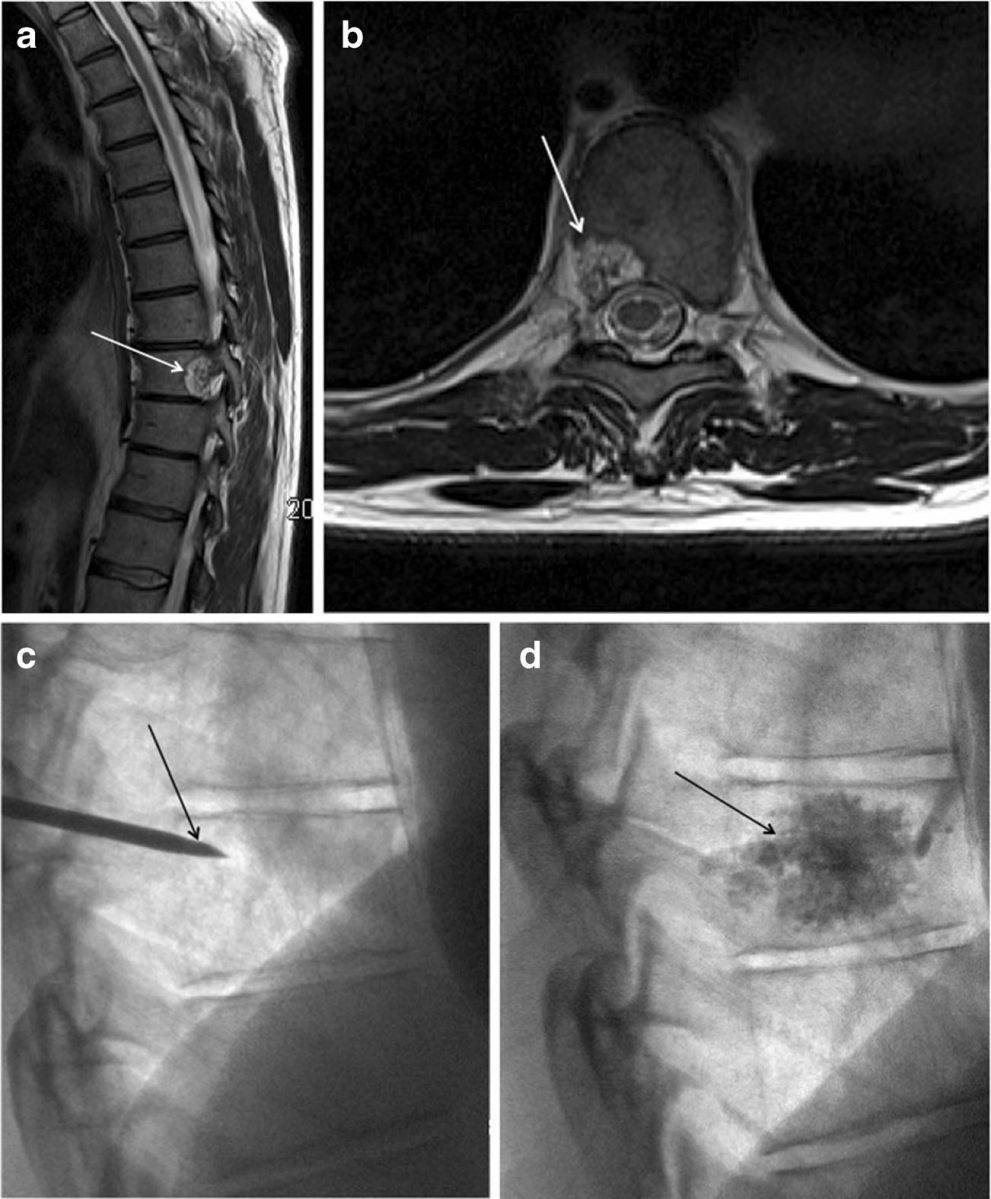
46-year-old man with a 2-year history of thoracic back pain without any neurological deficit. Sagittal (**a**) and axial T2 (**b**) weighted sequences show a lesion within the right side of the T9 vertebral body extending into the posterior elements. The lesion is T2-hyperintense. The decision was made to treat with vertebroplasty and perform an intraprocedural bone biopsy. Selected images from the procedure show a right transpedicular approach at T9 (**c**) with injection of methyl methacrylate into the lesion (**d**)

**Fig. 3:**
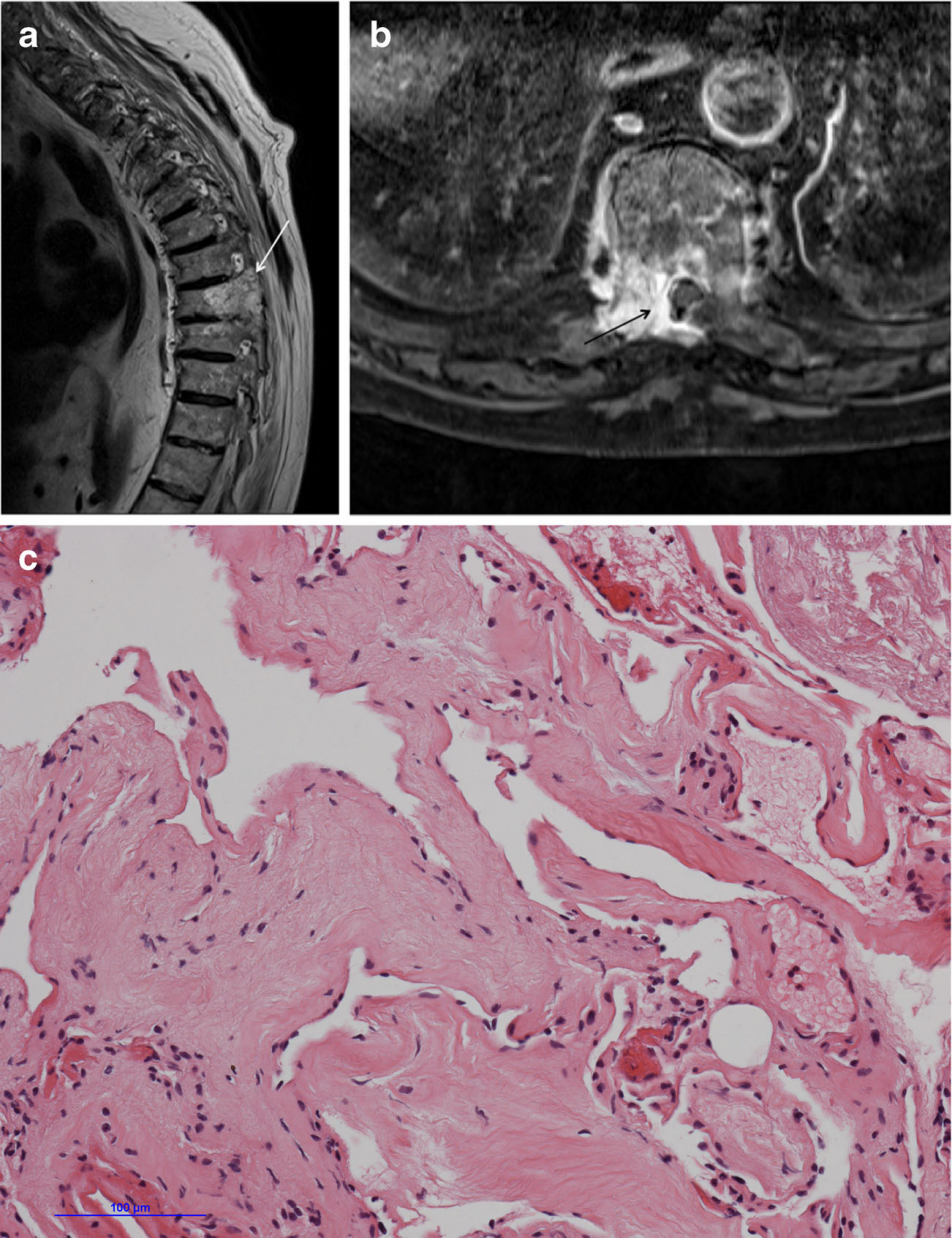
Case 1 from Table 1: 67-year-old man with history of previously excised spinal haemangioma and prostate cancer. MRI was performed following the development of new back pain. Sagittal T2-weighted sequence (**a**) shows abnormal signal in the right posterolateral vertebral body of T8, extending into the right pedicle, lamina and transverse process. There is an associated mass in the spinal canal, shown on axial short tau inversion recovery (STIR) sequence (**b**) at the level of T8. Haematoxylin and eosin stain of biopsied vertebral lesion (**c**) confirms a recurrent haemangioma, with no evidence of metastatic prostate carcinoma

**Fig. 4:**
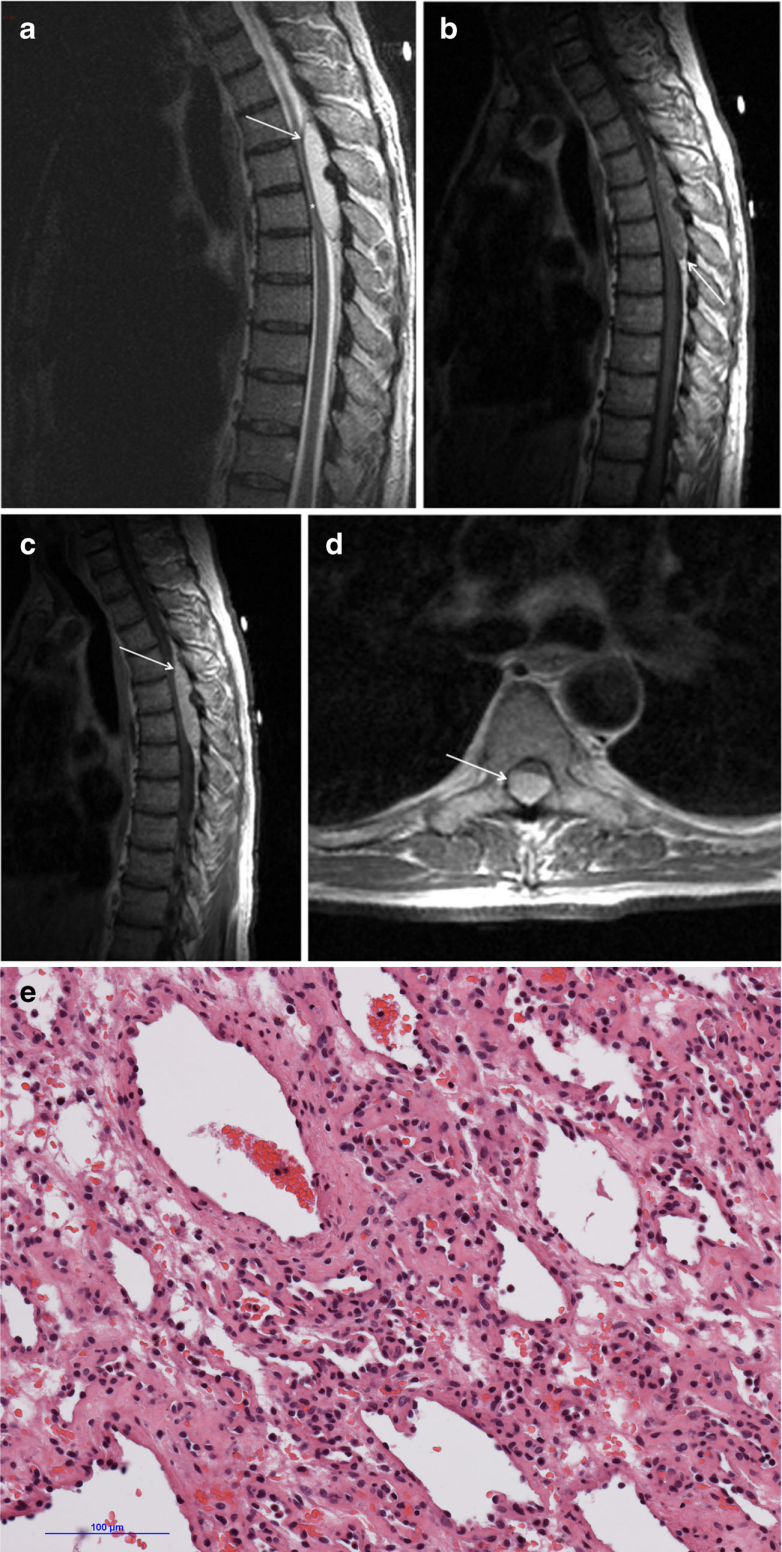
Case 2 from Table 1: 35-year-old man with gait disturbance and a sensory level at T6. Sagittal T2-weighted sequence (**a**) shows an extradural mass within the spinal canal posterior extending from T5 to T7. There is displacement of extradural fat on the non-contrast T1-weighted sequence (**b**). Sagittal (**c**) and axial T1-weighted post-contrast sequences (**d**) show avid enhancement of the mass. It displaces the cord anteriorly, and there is high signal intensity within the cord on the T2-weighted sequence (*asterisk*). Haematoxylin and eosin stain of excised extradural spinal mass (**e**) confirms a haemangioma

**Fig. 5:**
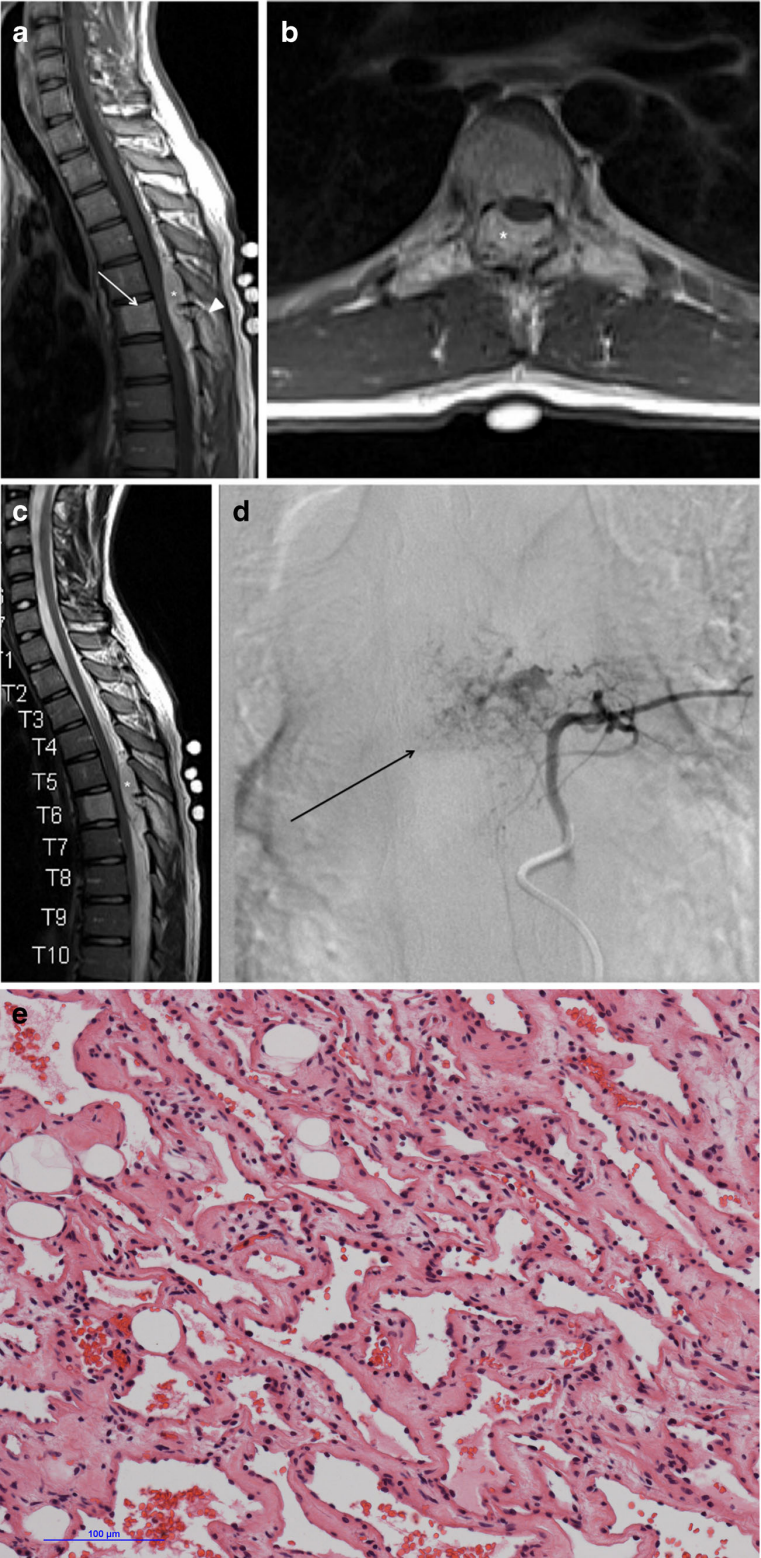
Case 3 from Table 1: 16-year-old boy with progressive paraesthesia and gait disturbance and a sensory level at T8. Sagittal (**a**) and axial (**b**) post-contrast T1-weighted sequences show abnormal high signal in the posterior two-thirds of the T6 vertebral body, extending into the posterior elements, including the pedicles, lamina and spinous process (*arrowhead*). There is an associated enhancing extradural mass posterior to the spinal cord extending from T5 to T7 (*asterisk*). The cord is displaced anteriorly. There is subtly increased signal intensity within the cord on the sagittal T2-weighted sequence (**c**) at the level of T6. Selective angiography of left T6 lumbar artery shows enhancement of the T6 vertebra and associated soft tissue mass (**d**). Haematoxylin and eosin stain of excised extradural spinal mass (**e**) confirms a haemangioma

**Fig. 6:**
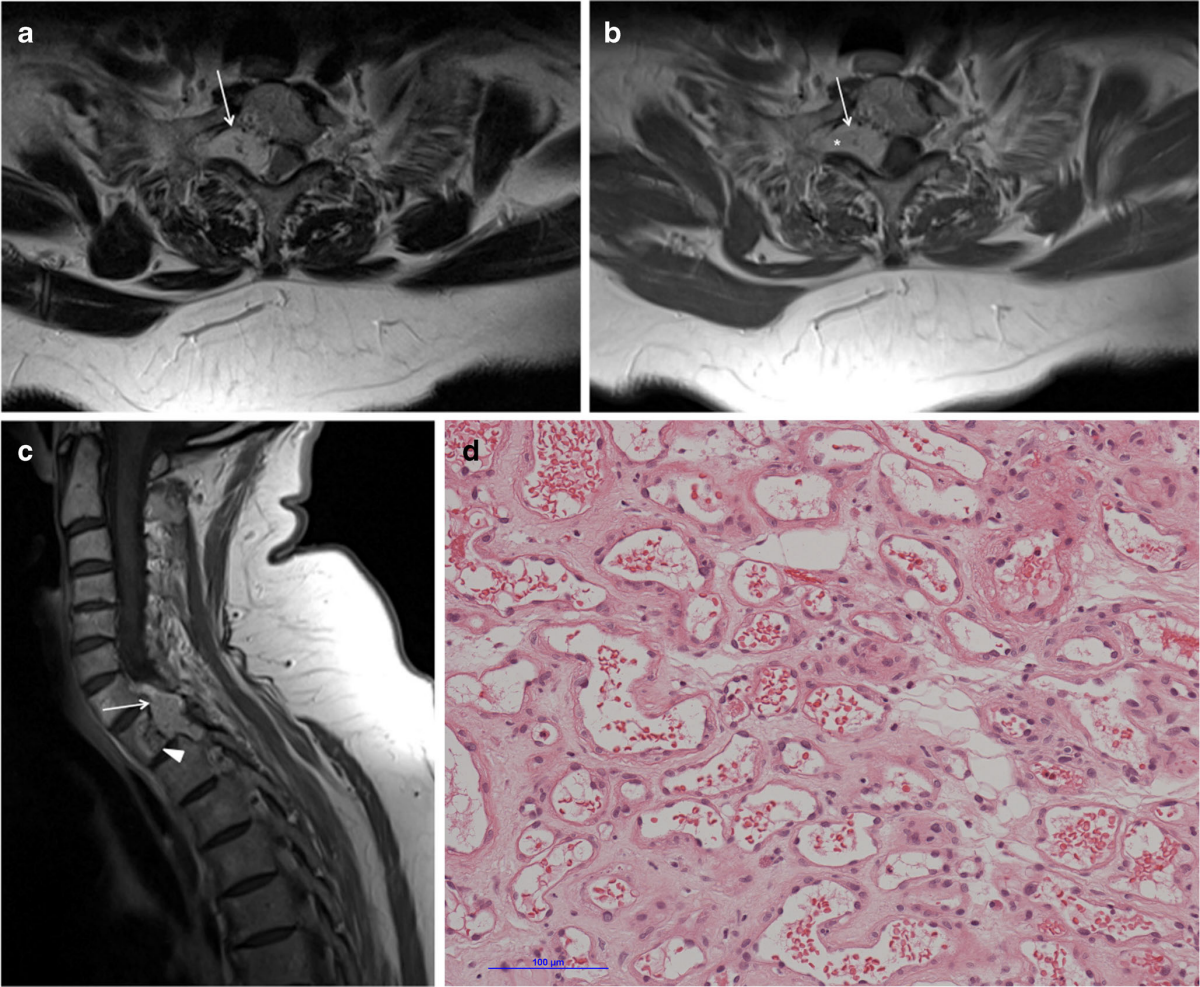
Case 4 from Table 1: 74-year-old woman with bilateral upper limb clumsiness and upper limb spasms. Axial T2-weighted sequence (**a**) and axial (**b**) and sagittal (**c**) post-contrast T1-weighted sequences show a 4-cm intradural mass at the level of T1, which is bright on the T2-weighted sequence and enhances homogeneously. It extends through the right neural foramen (*asterisk*). There is adjacent signal abnormality within the T1 vertebra and cortical destruction *(arrowhead*). Haematoxylin and eosin stain of excised intradural spinal mass with bone erosion (**d**) confirms a haemangioma

**Fig. 7:**
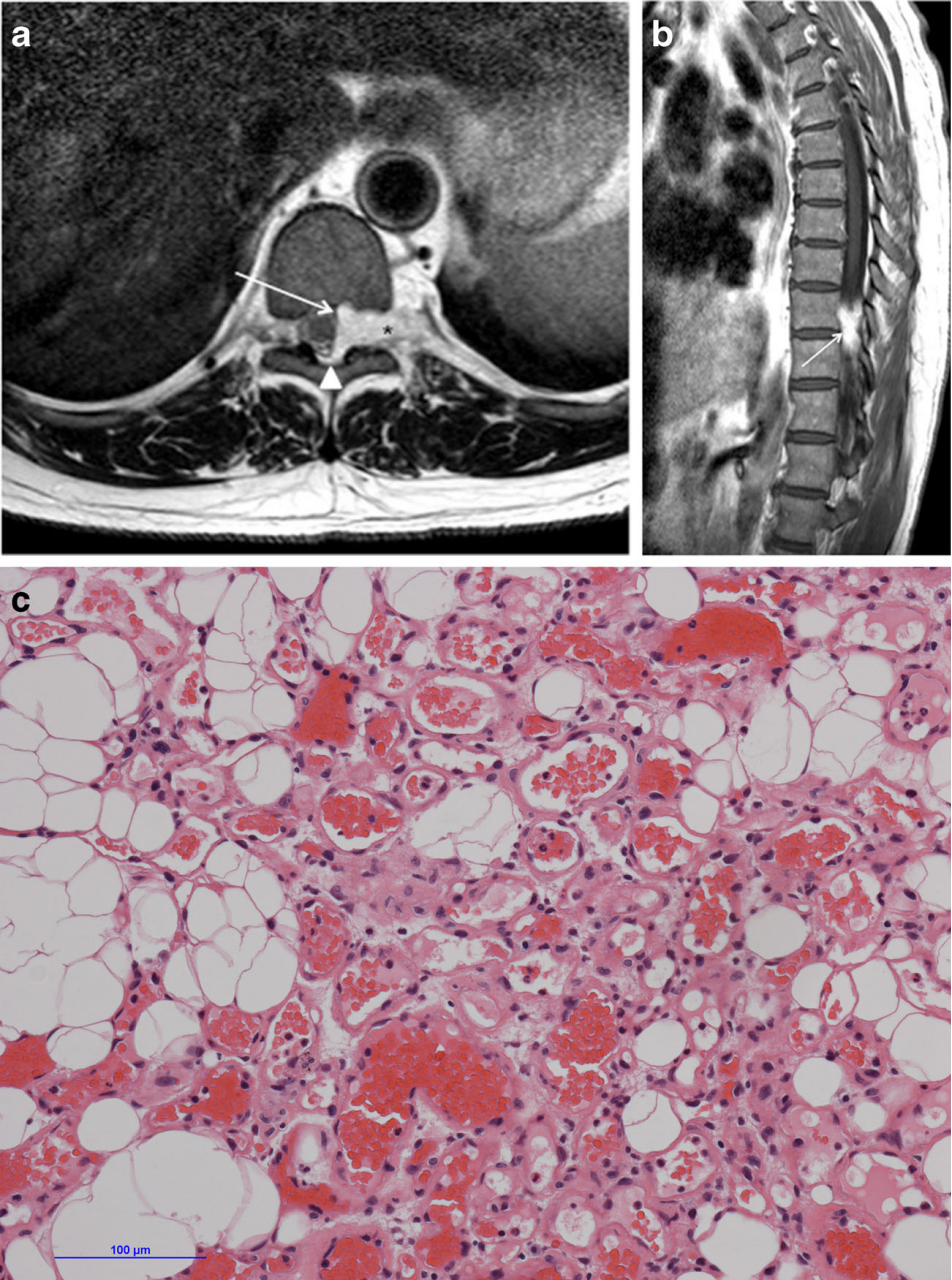
Case 5 from Table 1: 36-year-old man with progressive difficulty walking and a sensory level at T10. Axial T2-weighted sequence (**a**) shows an intradural extramedullary mass lateral to the spinal cord at the level of T10. The mass displaces the cord and extends out through the left neural foramen (*asterisk*). Note the preservation of the extradural fat (*arrowhead*). The mass enhances homogeneously on sagittal post-contrast T1-weighted sequence (**b**). Haematoxylin and eosin stain of excised intradural extramedullary spinal mass (**c**) confirms a haemangioma

**Fig. 8:**
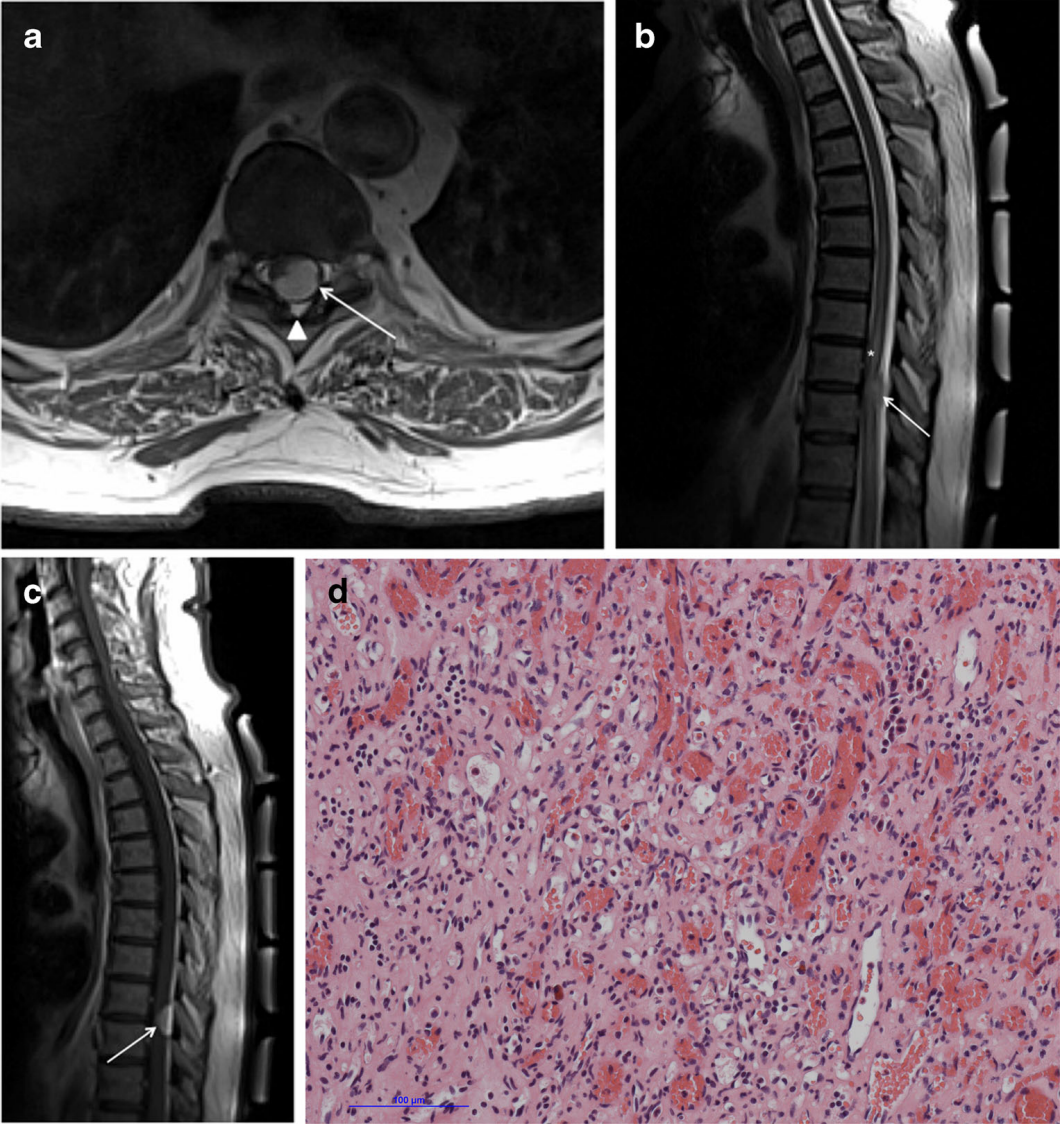
Case 6 from Table 1: 68-year-old woman with lower back pain and reduced power in both lower limbs. Axial (**a**) and sagittal (**b**) T2-weighted sequences show an intradural mass posterior to the spinal cord at the T9–T10 intervertebral disc space. There is high signal intensity within the cord (*asterisk*). Note again the preservation of the posterior extradural fat (*arrowhead*). The mass enhances homogeneously on sagittal post-contrast T1-weighted sequence (**c**). Haematoxylin and eosin stain of excised intradural spinal mass (**d**) confirms a haemangioma

A search of the neuropathology database at our institution was performed to identify all cases of pathologically proven spinal haemangiomas over the past 15 years. Six cases were identified with radiological–pathological correlation. The salient clinical, radiological and pathological features of each of these cases are outlined in Table 1 (Figs 1, 2, 3, 4, 5, 6, 7 and 8).

**Table 1:** Case series of spinal haemangiomas with radiological–pathological correlation

Case	Clinical history	Radiology	Outcome	Pathology
1	67-year-old man with history of a spinal haemangioma excised 14 years previously. Followed radiologically without evidence of recurrence. Subsequently diagnosed with prostate cancer 6 years later, treated with local radiotherapy and hormone therapy. Developed new thoracic back pain 2 years following this, without neurologic deficit.	MRI images (*a*) and (*b*) show extensive abnormal signal in the T8 vertebral body extending into the posterior elements, and a mass in the spinal canal at the level of T8.	Bone biopsy was performed to exclude prostate metastasis. Patient is currently being managed conservatively.	Haematoxylin and eosin stain of biopsied vertebral lesion (*c*) confirms a recurrent haemangioma with no evidence of metastatic prostate carcinoma.
2	35-year-old man with background of Henoch-Schonlein purpura and end-stage renal disease. Presented with a 1-week history of gait disturbance. Hyperreflexia and hypertonia on examination, with a sensory level at T6.	MRI images (*a*), (*b*), (*c*) and (*d*) show an enhancing extradural mass within the spinal canal extending from T5 to T7.	Underwent T6-T7 laminectomy and resection of spinal mass. At surgery the mass was noted to bleed significantly. Uncomplicated postoperative course with improved symptoms.	Haemotoxylin and eosin stain of excised extradural spinal mass (*e*) confirms a haemangioma.
3	16-year-old boy with 3-week history of progressive paraesthesia and gait disturbance. On examination had sensory level at T8.	MRI images (*a*) and (*b*) show abnormal high signal in the posterior two-thirds of the T6 vertebral body extending into the posterior elements, and an associated enhancing extradural mass posterior to the cord extending from T5 to T7.	Underwent preoperative embolisation followed by T5–T7 laminectomy and excision of the spinal mass. Improved neurological symptoms postoperatively but developed kyphosis at the level of the previous surgery, which required subsequent surgical fixation. A digitally subtracted image is shown from the embolisation procedure. Selective angiography of the left T6 lumbar artery shows enhancement of the T6 vertebra and associated soft tissue mass (*d*).	Haemotoxylin and eosin stain of excised extradural spinal mass (*e*) confirms a haemangioma.
4	74-year-old woman with 2-year history of bilateral upper limb clumsiness and episodic upper limb spasms	MRI images (*a*), (*b*) and (*c*) show an enhancing 4-cm intradural mass at the level of T1 extending through the right neural foramen. There is associated signal abnormality within the T1 vertebra and cortical destruction.	Underwent C7–T2 laminectomy and attempted resection of the spinal mass. The procedure was abandoned due to extensive intraoperative bleeding. The mass was subsequently embolised, and the patient underwent successful complete excision.	Haematoxylin and eosin stain of excised intradural spinal mass with bone erosion (*d*) confirms a haemangioma.
5	36-year-old man with progressive difficulty walking and diminished sensation in the lower limbs over 6 months. Hyperreflexia and reduced power (4/5) on examination, with sensory level at T10.	MRI images (*a*) and (*b*) show an enhancing intradural extramedullary mass at the level of T10 extending out through the left neural foramen.	Underwent T10 laminectomy and excision of spinal mass. Symptoms of myelopathy resolved postoperatively.	Haemotoxylin and eosin stain of excised intradural extramedullary spinal mass (*c*) confirms a haemangioma.
6	68-year-old woman with 8-week history of lower back pain with associated paraesthesia in the left lower limb. On examination, reduced power in both lower limbs (3/5 on left side and 4/5 on right side)	MRI images (*a*), (*b*) and (*c*) show an enhancing intradural mass at the T9–T10 disc space.	Underwent T8–T10 laminectomy and excision of spinal mass. Made a slow improvement postoperatively.	Haemotoxylin and eosin stain of excised intradural spinal mass (*d*) confirms a haemangioma.

### Osseous haemangiomas with extradural extension

In this discussion of spinal haemangiomas, we have included vertebral body haemangiomas with extradural extension and both intradural and extradural soft tissue haemangiomas.

Vertebral haemangiomas are the most common benign vertebral neoplasm, having been found in 11 % of spines at autopsy [1]. They are usually primarily within the vertebral body, less commonly involving the pedicles or posterior elements. They are most frequently located in the lower thoracic and lumbar vertebrae. Multiple haemangiomas occur in roughly one-third of cases. Haemangiomas are more common in women, with peak occurrence in the fourth to sixth decades of life. Although typically small and slow-growing, an aggressive radiologic growth pattern may be evident, with features that include bone expansion, extension into posterior elements, extraosseous extension, disturbance of local blood flow and compression fractures. Aggressive haemangiomas can result in local or radicular pain secondary to vertebral body collapse or encroachment into the spinal canal and cord compression from extradural haemorrhage or soft-tissue extension [2]. Paradoxically, symptomatic haemangiomas may show no radiologic signs of aggression, and aggressive haemangiomas may be asymptomatic [3]. Only 0.9–1.2 % of all vertebral haemangiomas are symptomatic [4], with pain or radiculopathy, myelopathy and paralysis [5] the most common presenting features. Extraosseous extension is more likely in aggressive and atypical cases.

### Pathology

Vertebral haemangiomas consist of mature thin-walled blood vessels and sinuses lined by flattened or attenuated endothelium. Occasionally the endothelium may have a hobnail appearance. Vessels are interspersed among longitudinally oriented trabeculae of bone and fatty matrix, and may actually cause resorption of underlying bone and thickening of the remaining trabeculae. Cavernous and capillary haemangiomas are the most prevalent pathological subclasses of vertebral haemangiomas.

### Imaging appearance

Vertebral haemangiomas rarely create a diagnostic dilemma. On plain film and on sagittal or coronal CT, they have a classical parallel striated appearance in the vertebral body (‘striped pyjamas’ or ‘corduroy cloth’ appearance), and on axial CT they have a spiculated or spotted appearance as the lesions are being imaged in cross-section (‘polka dot’ appearance). These striations are secondary to vascular channels intermingled with thickened vertical trabeculae which preserve the function of the vertebra to withstand an axial load. At MR imaging, vertebral haemangiomas demonstrate high signal intensity on T1- and T2-weighted images. Autopsy specimens of vertebral haemangiomas have been examined with MRI and correlated histologically [6]. The degree of high signal intensity on T1- and T2-weighted imaging is related to the proportion of the lesion surface area occupied by adipocytes, vessels and oedema. Typical haemangiomas are hyperintense on T1 because of their fatty stroma, and hyperintense on T2 because of their vascular components. They demonstrate variable enhancement, with less enhancement seen in lesions with a larger fatty component. Haemangiomas typically retain some high signal on fat-suppressed sequences, e.g. short tau inversion recovery (STIR); because of their vascular elements, the degree of high signal on STIR imaging depends on the balance of fat and vascular components.

Atypical haemangiomas such as epithelioid haemangioma or haemangioendothelioma may demonstrate a mixed pattern, with heterogeneous signal intensity on T1- and T2-weighted MR imaging that reflects the presence of inflammatory infiltrate and the absence of fat. A haemangioma is more likely to show aggressive behaviour when its stroma contains less fat and is more vascular. The presence of low signal on T1-weighted images and high signal on T2-weighted images on MRI, avid contrast enhancement, the presence of cortical erosion, soft tissue stroma between the osseous trabeculae on CT, the presence of extradural soft tissue, expansion to involve the posterior elements, invasion of the spinal canal, and encroachment of the spinal cord are all radiological features of aggressiveness [7]. Extraosseous extension into the extradural space is an uncommon feature associated with increased symptoms and aggressiveness. Atypical or aggressive haemangiomas can simulate vertebral metastases, focal myelomatous infiltration or lymphoma. Lesion signal intensities can be unhelpful, as all of these pathologies may be low signal on T1-weighted images and high signal on T2-weighted and STIR images. The absence of change on pre- and post-treatment MRI may also not be conclusive, as multiple myeloma lesions may not revert to normal marrow signal intensity until nearly 5 years post-treatment [8]. Lesion morphology, in particular the presence of coarsened trabeculae, can be helpful for differentiating haemangiomas from malignant lesions. CT is more sensitive to the characteristic osseous remodelling of haemangiomas than MRI, and may be required for problem-solving [2].

Spinal angiography is not routinely used for the diagnosis of vertebral haemangiomas, and should be reserved only for cases requiring a thorough evaluation prior to biopsy, embolisation or surgical intervention. At angiography, haemangiomas demonstrate variable enhancement. Along with lesion characterisation, angiography also allows localisation of the origin of spinal arteries.

### Treatment and prognosis

Treatment of vertebral haemangiomas is usually only necessary when they are symptomatic with neurological deficit or pain. Radiotherapy may have a therapeutic role in selected cases [9], and radiotherapy after surgical decompression may be beneficial [10]. Repeated radiotherapy for recurrence may rarely induce malignant transformation to haemangiosarcoma [11]. Percutaneous injection of ethanol directly into the haemangioma under fluoroscopic or CT guidance may be effective for symptom relief, provided that the dose is less than 15 ml [12]. Controversy exists regarding the safety of this technique, as there have been reports of infrequent neurological and cardiovascular complications [12–14]. Endovascular embolisation is another treatment option, performed either as standalone treatment or preoperatively. Endovascular embolisation alone can be used successfully to treat pain. However, if used as the sole treatment for cord compression, it may fail either because of the presence of tortuous vessels, because the arterial supply to the cord arises from feeding intercostal vessels, or due to the presence of tiny feeders that cannot be occluded [15, 16]. Selective spinal angiography is essential for demonstrating arterial supply to the haemangioma and for determining the contribution from the artery of Adamkiewicz, which, if unintentionally embolised, may cause infarction of the cord. Surgical intervention is generally reserved for patients with neurological complications. Cord decompression can be accomplished with laminectomy or vertebrectomy. Open procedures can be complicated by major haemorrhage, although preoperative embolisation reduces expected intraoperative blood loss [15]. A recent report of 31 patients who underwent vertebroplasty for the treatment of painful vertebral haemangiomas without neurological deficit found vertebroplasty to be feasible, efficacious and safe [17]. It has been postulated that haemangiomas might be more susceptible to cement leakage due to the presence of abnormal vascularisation, high-flow dilated blood channels and the formation of vertebral venous neoanastomoses. Vertebroplasty has been shown to be an effective therapy even in cases of extradural extension [3].

### Non-osseous extramedullary spinal haemangiomas

In addition to vertebral bone haemangiomas that may extend into the extradural space, pure soft-tissue non-osseous extramedullary spinal haemangiomas may be either extradural or intradural. Precise determination of the radiologic relationships between the dura and the haemangioma can be challenging. If the extradural fat is preserved, the lesion is likely to be intradural. Occasionally the correct topography cannot be determined radiologically, and may be confirmed only during surgical resection.

### Cavernous haemangiomas

Cavernous haemangiomas are typically extradural in location and usually associated with bone involvement, i.e. representing extension from vertebral haemangiomas into the canal. However, they can occur without bone involvement [18]. They comprise 4 % of all extradural tumours and 12 % of all haemangiomas within the canal [19]. The most frequent clinical presentation is progressive myelopathy. Acute onset of symptoms is rare, and is typically caused by haemorrhage. An intradural-located cavernous haemangioma has been reported, causing recurrent subarachnoid haemorrhage [20].

### Pathology

Cavernous haemangiomas of the spinal canal consist of multiple thin-walled vascular channels in collagenous connective tissue lined by a single layer of endothelial cells. As previously mentioned, the hallmark of haemangiomas is the presence of a distinct vessel of origin, i.e. capillary, cavernous or venous. Angiolipomas, in comparison, are made up almost entirely of fat, with a sparse vascular component.

### Imaging features

MRI shows a lobular extradural mass that is not anatomically connected to the neighboring intervertebral disc or the exiting nerve. Typically, the mass exhibits high signal intensity on T2-weighted sequences, and demonstrates strong homogeneous enhancement post-contrast administration [21]. In addition to these more common features, T1 hyperintensity, multi-segment involvement and ovoid shape may be a clue in the differential diagnosis of a spinal extradural haemangioma. Differential diagnosis for extradural haemangiomas includes disc herniation, extradural haematoma, extradural metastases and extramedullary haematopoiesis [18]. If the diagnosis is being considered, spinal angiography may be useful for differentiating haemangiomas from non-vascular tumours, thus reducing the risk of intraoperative bleeding.

### Treatment and prognosis

Surgical resection is the treatment of choice for extradural haemangiomas [21]. Misinterpretation or failure to consider haemangioma in the imaging differential may result in unexpected intraoperative haemorrhage due to the high vascularity of these lesions. Incomplete surgical removal of an extradural haemangioma because of intraoperative bleeding or minimal exposure during disc surgery can result in persistent symptoms or recurrence. Adjunct radiotherapy has been performed in cases of subtotal excision of an extradural cavernous haemangioma [22].

### Capillary haemangiomas

Capillary haemangiomas of the spinal canal are very rare, and are typically intradural and extramedullary in location. They may arise from the nerve root vessels in the cauda equina, the inner surface vessels of the dura or the pial surface vessels of the spinal cord. There have been approximately 36 cases of intradural extramedullary capillary haemangiomas reported to date [23]. Similar to other soft tissue tumours of the spinal canal, capillary haemangiomas may present with progressive myelopathy or radiculopathy. There is also a significant risk of bleeding, resulting in sudden neurologic deterioration, with associated haematomyelia or subarachnoid haemorrhage [5].

### Pathology

Capillary haemangiomas are histologically characterised by abnormal small, closely packed capillary vessels lined by flattened endothelium separated by a collagenous stroma. Similar to cavernous haemangiomas, they show a clear demarcation from the surrounding tissue, unlike other vascular lesions such as arteriovenous malformations, which interdigitate into the tissue.

### Imaging features

MR imaging demonstrates a well-demarcated mass in the extramedullary space [24]. The signal intensity of the mass is isointense to the cord on T1-weighted images and hyperintense relative to the cord on T2-weighted images, and demonstrates strong enhancement following administration of gadolinium. As these signal characteristics are similar to those of other intradural extramedullary masses, such as meningiomas or nerve sheath tumours, it can be close to impossible to differentiate haemangiomas from these entities on MRI. The presence of subtly enlarged retromedullary abnormal vessels on contrast MR images or areas of signal void on T2-weighted imaging are suggestive of haemangiomas, and preoperative spinal angiography should be considered [25]. Differential diagnoses to consider apart from meningiomas and nerve sheath tumours include lymphoma, sarcoidosis and rare tumours such as haemangioendothelioma.

### Treatment and prognosis

The natural history of capillary haemangiomas has not been well described, but there is a significant risk of bleeding and enlargement, with resultant neurologic deterioration. The current treatment of intradural capillary haemangiomas consists of total excision. Most reported cases have been successfully resected without major intraoperative bleeding. To avoid bleeding, preoperative angiography and embolisation can be considered. The role of radiation therapy has not yet been investigated for the treatment of capillary haemangiomas. If total excision is not achieved, regular follow-up MRI examinations are warranted [26].

## Conclusion

While vertebral body haemangiomas are a common and often incidental and unimportant imaging finding, they may display aggressive features and cause significant neurologic symptoms and signs necessitating treatment. One such aggressive feature on imaging is the extension of the haemangioma into the extradural space. Nonetheless, primary extraosseous extradural and intradural extramedullary haemangiomas are rare, having previously been published only in isolated case reports. Magnetic resonance imaging plays a pivotal role in characterising spinal haemangiomas, although imaging characteristics may be diverse, and spinal angiography can be used to differentiate haemangiomas from non-vascular lesions. Radiological awareness of extramedullary haemangiomas is important, as they can be considered as a differential diagnosis for extramedullary masses.
